# Expanding the scope of PI3K-δ inhibition: Leniolisib treatment in PRKCD deficiency

**DOI:** 10.70962/jhi.20250203

**Published:** 2026-04-20

**Authors:** Lorenzo Lodi, Valentina Guarnieri, Matilde Peri, Manuela Baronio, Silvia Ricci, Clementina Canessa, Francesca Lippi, Marta Voarino, Elisa Calistri, Laura Pisano, Anna Perrone, Grazia Fenu, Giuseppe Indolfi, Vassilios Lougaris, Rebecca A. Marsh, Chiara Azzari

**Affiliations:** 1 Pediatric Immunology Unit, Meyer Children’s Hospital IRCCS, Florence, Italy; 2 University of Florence, Florence, Italy; 3 Department of Health Sciences, University of Florence, Florence, Italy; 4 Department of Clinical and Experimental Sciences, Pediatrics Clinic, Azienda Socio-Sanitaria Territoriale Spedali Civili di Brescia, University of Brescia, Brescia, Italy; 5 Laboratory of Immunology and Molecular Microbiology, Meyer Children’s Hospital IRCCS, Florence, Italy; 6 Pediatric Radiology Unit, Meyer Children’s Hospital IRCCS, Florence, Italy; 7 Pediatric Pulmonary Unit, Meyer Children’s Hospital IRCCS, Florence, Italy; 8 Pediatric Liver Unit, Meyer Children’s Hospital IRCCS, Florence, Italy; 9 Division of Bone Marrow Transplantation and Immune Deficiency, Cincinnati Children’s Hospital Medical Center, Cincinnati, OH, USA; 10 Department of Pediatrics, University of Cincinnati, Cincinnati, OH, USA; 11 Pharming Healthcare, Inc., Warren, NJ, USA

## Abstract

Leniolisib, a selective phosphoinositide 3-kinase δ (PI3Kδ) inhibitor, is approved in several countries for the treatment of activated PI3Kδ syndrome (APDS) in patients 12 years of age and older. We report the first successful compassionate use of leniolisib in another inborn error of immunity, protein kinase C δ deficiency. In addition to infectious complications, the 14-year-old patient experienced lymphoproliferation in the form of splenomegaly, lymphadenopathy, and thymic hyperplasia; trilineage cytopenia; multiple forms of autoimmunity; and interstitial lung disease. Decision to initiate treatment with leniolisib was based on multiorgan-disease progression, lack of therapeutic alternatives, molecular evidence, overlap with APDS manifestations, and mammalian target of rapamycin hyperactivity. We observed improvement in lymphoproliferation, cytopenias, hepatic cytolysis, skin manifestations, pulmonary function, favorable changes in immunophenotypes, and no known drug-related adverse events. This experience supports expanding Leniolisib’s potential indications to appropriately selected patients and conditions. Broader repurposing strategies for targeted therapies in diseases involving dysregulated PI3K signaling should be systematically evaluated in clinical trials.

## Teaser

Lodi et al. report leniolisib use, a PI3Kδ inhibitor approved for APDS, in a teenager with protein kinase C δ deficiency and progressive multiorgan immune dysregulation. Treatment reduces lymphoproliferation and cytopenias and improves lung and skin disease, showing no drug-related adverse events. This experience supports expanding Leniolisib’s potential indications to appropriately selected patients and conditions.

## Introduction

Protein kinase C δ (PKCδ) deficiency is an inborn error of immunity (IEI) caused by autosomal recessive variants in *PRKCD* that result in PKCδ loss-of-function (1, 2, 3). PKCδ is a ubiquitously expressed serine-threonine kinase involved in cell proliferation, differentiation, and apoptosis. Once activated, PKCδ is modulated by phosphoinositide 3-kinase (PI3K) and mammalian target of rapamycin (mTOR) (4).

**Figure 1.**
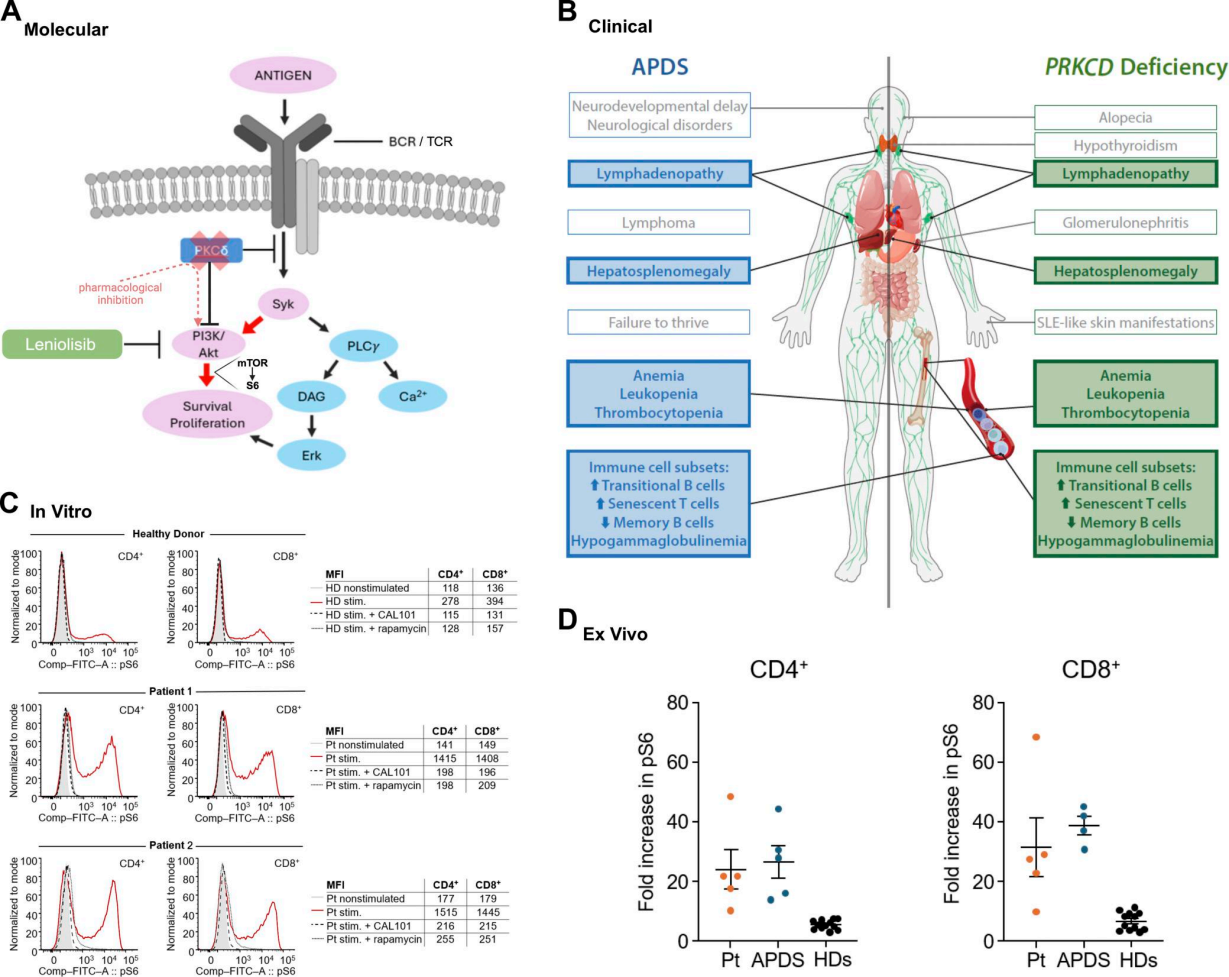
**Rationale for leniolisib treatment. (A)** Potential interactions of PKCδ and PI3K pathways. **(B)** Clinical comparison of APDS and PRKCD deficiency. **(C)** Representative pS6 flow cytometry assay of stimulated and nonstimulated CD4^+^ and CD8^+^ T cells from an HD (top), patient 1 (index case described in the report), and patient 2 (his affected sibling). Stimulated cells were also pretreated with CAL101 (idelalisib, black dashed line) to inhibit PI3Kδ signaling or rapamycin (grey dashed line) to inhibit mTOR signaling. **(D)** Summarized data showing CD4^+^ and CD8^+^ T cells from the index patient and patients with APDS display increased pS6 upon in vitro stimulation compared with HDs. BCR, B cell receptor; Comp-FITC-A, compensation control fluorescein isothiocyanate; DAG, diacylglycerol; ERK, extracellular signal-regulated kinase; HD, healthy donor; MFI, mean fluorescence intensity; PLCγ, phospholipase C γ; PRKCD; protein kinase C δ gene; Pt, patient; SLE, systemic lupus erythematosus; stim; stimulated; Syk; spleen tyrosine kinase.

Although the interaction between PKCδ and PI3K remains incompletely defined, PKCδ may inhibit spleen tyrosine kinase, influencing downstream PI3K-dependent signals in B lymphocytes (4, 5) (Fig. 1 A). Furthermore, previous studies have shown that pharmacological inhibition of PRKC activity leads to AKT hyperphosphorylation, suggesting that the PI3K/AKT signaling pathway is negatively regulated by PKC (6). PKCδ also impacts the function of other immune cells (4). Knockout (Prkcd^−/−^) mice have disrupted immune homeostasis, resulting in autoimmunity, lymphoproliferation, and B cell infiltration (3, 7, 8).

Since 2013, PKCδ deficiency has been reported in 21 patients (Table 1, 3, 9, 10, 11, 12, 13, 14, 15, 16, 17), revealing a diverse clinical phenotype paralleling the murine model. Patients most commonly present with early-onset and often severe autoimmunity, particularly lupus-like disease with multiorgan involvement, including immune-mediated cytopenias, nephritis, and systemic inflammation. Lymphoproliferative features such as persistent lymphadenopathy and splenomegaly are frequently observed. Recurrent and sometimes severe infections are also reported, involving both common bacterial pathogens and opportunistic organisms. Most cases present with a combination of autoimmunity, lymphoproliferation, and susceptibility to infections, while some exhibit a singular phenotype. Manifestations overlap with other disorders, including autoimmune lymphoproliferative syndrome, monogenic systemic lupus erythematosus, common variable immunodeficiency, and chronic granulomatous disease (3). Accordingly, therapeutic strategies vary (Table 1) and mainly include immunomodulatory agents such as mTOR inhibitor rapamycin. Hematopoietic stem cell transplantation (HSCT) was performed in two cases (9).

**Table 1.** PKCδ deficiency reported in the literature

Reference	Age at onset	Sex	Ethnicity	Genetic variant	Initial presentation	Infections	Lymphoproliferation	Autoimmunity	Laboratory	Treatment(s)	Age at last follow-up	Alive/dead
Salzer et al., 2013, PT1 (2)	Infancy	M	Turkish	c.1352+1G>A	Recurrent infections	URTI (i.e., pneumonia), LRTI, UTIs, gastroenteritis otitis media, and herpes virus viremia	Hepatosplenomegaly and lymphadenopathy	Nephrotic syndrome, nonspecific reactive follicular hyperplasia, relapsing polychondritis, hypothyroidism, antiphospholipid syndrome, and SLE	Progressive reduction in B cells, reduced memory B cells, increased CD21^low^ B cells, low IgG levels, and elevated IgA and IgM levels	Steroids, rituximab, MMF, IVIG, enalapril, anticoagulants, and thyroid hormone replacement	12 years	Alive
Belot et al., 2013, PTV1, V3, V4 (10)	10 years	F	White, of Northern European extraction	c.1528G>A	Discoid lupus rash and arthritis	None reported	Hepatomegaly	Alopecia, lupus nephritis, arthritis, chronic cutaneous lupus, WHO type 2 glomerulonephritis with severe nephrotic syndrome, chronic renal failure, SLE, and CNS vasculitis	Lymphopenia (B and T cells), positive ANAs (1:640) and anti-dsDNA antibodies, low C3 and C4, and elevated IgA	Steroids, hydroxychloroquine, Azathioprine, methotrexate, and MMF	24 years	Alive
	3 years	F	White, of Northern European extraction	c.1528G>A	Diffuse lymphoproliferation and AIHA	*P. aeruginosa* septic shock	Lymphadenopathy, multiple compressive adenopathy with mediastinal involvement and hepatosplenomegaly	SLE, AIHA, serositis, pancreatitis, ITP, lupus rash, and lupus nephritis	Lymphopenia, but increased CD3^+^ T cells, undetectable double-negative TCRα/β T cells, positive ANAs (1:640) and anti-dsDNA antibodies, and low C3 and C4	Immunosuppressive treatments	8 years	Died at age 13 years (end-stage kidney failure complicated by *Pseudomonas* sustained septic shock)
	6 years	M	White, of Northern European extraction	c.1528G>A	Lupus nephritis	​	None	Renal flare with malar rash and arthritis SLE	Increased immature and naive B cells, reduced memory B cells, positive ANAs, and anti-dsDNA antibodies	Steroids (prednisolone) and MMF	13 years	Alive
Kuehn et al., 2013, PT1 (1)	3 years	M	Hispanic	c.1840C>T	Recurrent otitis, sinusitis, persistent lymphadenopathy, hepatosplenomegaly, and intermittent fevers	Recurrent otitis media/sinusitis, and persistent EBV	Hepatosplenomegaly and mediastinal lymphadenitis (with superior vena cava syndrome)	SLE and AIHA	CD5^+^ majority B lymphocytosis; reduced class-switched memory B cells; elevated DNT αβ and DNT γδ; low levels of NK cells and NK cell cytolytic activity; increased ESR, CRP, ALT, and AST; hypergammaglobulinemia; positive ANAs; anti-RNP; anti-Smith; anti-SSA	Steroids (prednisone) and sirolimus	7 years	Alive
Kiykim et al., 2015, PT6 (11)	Infancy	M	Turkish	c.742G>A	Recurrent fever	Pneumonia, gastroenteritis, and CMV	Hepatosplenomegaly and lymphadenopathy	SLE	Naive majority B lymphocytosis and CD21^low^, reduced levels of switched memory B cells, increased IgM, and reduced NK functionality	Ganciclovir, IVIG, topical steroids, hydroxychloroquine, IVIG, and prophylactic antibiotics	3.5 years	Alive
Lei et al., 2018, PT II-1, PT II-2, and PT 11-3 (12)	1 year	F	Endogamous Pakistani	c.1294G>T	Constitutional symptoms (intermittent fever, night sweats, fatigue), severe thrombocytopenia, photosensitive and petechial rash, scarring alopecia, and hepatosplenomegaly	NR	Splenomegaly and lymphadenopathy	SLE (acuta cutaneous lupus, oral/nasal ulcers)	AIHA, thrombocytopenia, leukopenia, positive ANA (1:1,280), anti-dsDNA, antiphospholipid antibody, low complement, elevated CRP and ESR, elevated IgG, and low levels of C4	Steroid, rituximab (discontinued for adverse reaction), IVIG, hydroxychloroquine, Azathioprine, and Ofatumumab	NR	Alive
	1 year	F	Endogamous Pakistani	c.1294G>T	Constitutional symptoms (intermittent fever, night sweats, fatigue), severe thrombocytopenia, photosensitive and petechial rash, scarring alopecia, hepatosplenomegaly	NR	Hepatosplenomegaly, lymphadenopathy	SLE (acute cutaneous lupus, serositis, and renal impairment)	AIHA, thrombocytopenia, leukopenia, slight neutropenia, positive ANA (1:1,280), anti-dsDNA, antiphospholipid antibody, and low complement, elevated CRP and ESR, elevated albumin/creatinine ratio, hypergammaglobulinemia, and low levels of C4	Steroid, IVIG, rituximab (discontinued for adverse reaction),h ydroxychloroquine, Azathioprine, and Ofatumumab	NR	Alive
	26 mo	M	Endogamous Pakistani	c.1294G>T	Constitutional symptoms (intermittent fever, night sweats, fatigue), severe thrombocytopenia, photosensitive and petechial rash, scarring alopecia, and hepatosplenomegaly	NR	Hepatosplenomegaly	SLE (acute cutaneous lupus)	AIHA, thrombocytopenia, positive ANA (1:1,280), anti-dsDNA, anti-Smith antibody, and low complement	Steroid (IV methylprednisolone), IVIG, and rituximab	NR	Alive
Sharifinejad et al., 2020, PT10 (13)	Infancy	M	Afghan	c.1293_1294insA	Recurrent oral candidiasis, nonsevere upper respiratory and gastrointestinal tract infections, and food allergy	Pneumonia, oral candidiasis, and recurrent gastroenteritis	Hepatosplenomegaly and lymphadenopathy	SLE	Increased DNT, reduced NK number, increased IgG, and nonprotective antidiphtheria	Corticosteroids, antibiotic prophylaxis	13 years	Alive
Neehus et al., 2021, PT13-17 (14)	1 year	F	Turkish	c.571+2dup	BCGosis, fever, left axillary, retroauricular, and cervical lymphadenopathies	BCGosis, *Salmonella* bacteremia, *Staphylococcus aureus* lymphadenitis, and recurrent gingivitis	Lymphadenopathy and hepatosplenomegaly	SLE	Elevated naive B cells and reduced B memory, with elevated CD21^low^ and transitional cells, hypogammaglobulinemia, ANA+, and anti-dsDNA+	Steroids IVIG Antimycobacterial (ceftriaxone); antibiotic prophylaxis (amoxicillin)	14 years	Alive
	7 mo	M	Turkish	c.1384C>T	Fever, diarrhea, hepatosplenomegaly, failure to thrive, left axillary lymphadenopathy with purulent discharge, and BCGitis	BCGitis, recurrent gastroenteritis	Hepatosplenomegaly	AIHA	Impaired DHR test and ANA+	Steroids Isoniazid Rifampicin	10 years	Alive
	7 mo	M	Turkish	c.1384C>T	BCGitis	BCGitis, recurrent gastroenteritis and shingles	Lymphadenopathy and hepatosplenomegaly	AIHA	Impaired DHR test and ANA+	Steroids Isoniazid Rifampicin IVIG	7 years	Alive
	1 year	F	Irani	c.642del	BCGitis	BCGitis	Hepatosplenomegaly and lymphadenopathy	None	Impaired DHR test and nonprotective antibody responses to tetanus and diphtheria	Isoniazid Rifampicin Azithromycin	3 years	Alive
	NR	M	Irani	c.642del	Asymptomatic	None	None	None	Impaired DHR test	No treatment	6 years	Alive
Gu et al., 2021, PT16-17 (15) and Jefferson et al., 2023 (3)	6 y	M	Chinese	c.36T>G	Lymphadenopathy, splenomegaly, and recurrent infections	NR	Splenomegaly and lymphadenopathy	Pancytopenia	Positive antibodies for EBV, CMV, herpes simplex virus, and rubella viruses, increased TCRαβ DNT, increased CD8^+^ T cells and IgG, and low levels of NK cells	Steroids, IRT, and sirolimus	10 years	Alive
	NR	F	Chinese	c.36T>G	Lymphadenopathy, splenomegaly, and anemia	NR	Splenomegaly and lymphadenopathy	Anemia	NR	NR	NR	NR
Yang et al., 2022, PT18 (18)	5 mo	F	Chinese	c.661C>T	Recurrent fever, respiratory tract infections, and lymphadenopathy	URTIs and BCGosis	Multiple lymphadenopathy (abdominal mesenteric lymph nodes invading the abdominal wall), bone marrow hyperplasia, and hepatosplenomegaly	NR	Increased DNT, elevated CD8^+^ T cells, low levels of CD4^+^ T cells, and CRP positive	Isoniazid, rifampicin, ethambutol, sirolimus, IFN-γ, and antibiotics	8 years	Alive
Neehus et al., 2022, PT19 (19)	2 years	F	American	c.285C>A/c.376C>T	URTI, lymphadenitis	*Burkholderia cepacia* and *Mycobacterium lentiflavum* lymphadenopathy	Infectious lymphadenopathy	None	Low levels of CD4^+^ T cells, decreased relative proportions of CD27^+^IgD^+^ and CD27^+^IgD^−^ memory B cells, and abnormally low DHR test	Cotrimoxazole, isoniazid, and Rifampicin	5 years	Alive
Roderick et al., 2023, PT20-21 (9)	9 mo	M	British	c.788-2A>G/c.571C>T	*Listeria* meningitis	*Listeria* meningitis, *Enterococcus faecium* bacteremia, varicella and shingles, invasive *Candida* infection, recurrent episodes of *Achromobacter xylosoxidans* cervical lymphadenitis, and early-onset enterocolitis	Lymphoid hyperplasia and splenomegaly	SLE, AIHA, and ITP	Increased DNTs, low B cells with low IgG with high IgM, positive anticardiolipin antibodies, Coombs positive, and positive thyroid peroxidase antibodies	Steroids, IVIG, rituximab, Sirolimus, Hydroxychloroquine, HSCT, and cyclosporine (for GVHD prophylaxis)	NR	Alive
	20 mo	F	British	c.788-2A>G/c.571C>T	Sepsis due to nontypeable *Haemophilus influenzae*	Early-onset enterocolitis	Lymphoid hyperplasia, splenomegaly	SLE	Increased DNTs, low B cells with low IgG and high IgM, positive anticardiolipin antibodies, and GAD antibody-positive	IVIG replacement therapy, rituximab, sirolimus (stopped because of oral ulceration), MMF, and hydroxychloroquine HSCT	8.5 years	Alive

AIHA, autoimmune hemolytic anemia; ALT, alanine aminotransferase; ANA, antinuclear antibody; AST, aspartate aminotransferase; BCG, Bacillus Calmette-Guérin; CMV, cytomegalovirus; CNS, central nervous system; CRP, C-reactive protein; C3; complement component 3; C4; complement component 4; DHR, dihydrorhodamine; DNTs, double-negative T cells; dsDNA, double-stranded DNA; EBV, Epstein-Barr virus; ESR, erythrocyte sedimentation rate; F, female; GAD, glutamic acid decarboxylase; GVHD, graft-versus-host disease; IFN-γ, interferon γ; IRT, immunoglobulin replacement therapy; ITP, immune thrombocytopenia; IVIG, intravenous immunoglobulin; LRTI, lower respiratory tract infection; M, male; MMF, mycophenolate mofetil; NK, natural killer; NR, not reported; PT, patient; RNP, ribonucleoprotein; SLE, systemic lupus erythematous; SSA, Sjögren syndrome type A; TCR, T cell receptor; URTI, upper respiratory tract infections; UTI, urinary tract infection; WHO, World Health Organization.

Leniolisib, a selective PI3Kδ inhibitor, is approved only for the treatment of activated PI3Kδ syndrome (APDS) in patients aged ≥12 years (18). APDS is characterized by increased AKT/mTOR signaling due to hyperactive PI3Kδ (19, 20). Inhibition restored immune cell function while attenuating lymphoproliferation (21, 22, 23). Here we present the first successful application of leniolisib for the treatment of *PRKCD* deficiency.

## Results

A 14-year-old male offspring of consanguineous parents (first cousins) was diagnosed with a pathogenic homozygous variant in *PRKCD* (NM_006254.4, c.1352+1G>A), leading to protein loss (1, 2). The parents were unavailable for segregation testing, but an older brother who experienced primary immunodeficiency with lymphoproliferation, autoimmunity, and predominant renal involvement had the same biallelic variant. The sibling clinical picture was characterized by membranous glomerulonephritis leading to chronic kidney disease in a solitary kidney, organizing pneumonia with splenomegaly and lymphadenopathy, and recurrent infections. Since early childhood, the proband experienced multiple hospitalizations for invasive bacterial and viral infections, including multidrug-resistant *Staphylococcus epidermidis* sepsis, human herpesvirus six encephalitis, recurrent *Mycoplasma pneumonia*e pneumonia, perforated *Pseudomonas aeruginosa* otitis media, and two episodes of respiratory syncytial virus pneumonia. Immunophenotyping revealed reduced memory B cells, increased transitional B cells, elevated immunoglobulin (Ig)M, and hypogammaglobulinemia that were treated with Ig replacement therapy and antibacterial prophylaxis. Moreover, he experienced autoimmunity and lymphoproliferation. In fact, by 12 years of age, he developed alopecia areata, trilineage cytopenia, autoimmune hepatitis, enteritis, splenomegaly, fluctuating lymphadenopathies, and thymic hyperplasia. Table 2 summarizes baseline findings.

**Table 2.** Clinical, immune, and radiological assessments pre-sirolimus and pre-leniolisib treatments

​	Pre-sirolimus assessment	Details pre-sirolimus	Pre-leniolisib assessment	Details pre-leniolisib
Age	12 years 6 mo	​	14 years 1 mo	​
Weight	29.4 kg	−2.39 SD	34.8 kg	−2.53 SD
Height	133.1 cm	−2.80 SD	147.9 cm	−2.04 SD
Clinical manifestation	Severe recurrent infections	Encephalitis—HHV-6 Sepsis—*S. aureus* Recurrent otitis—*P. aeruginosa* Pneumonia RSV—*M. pneumoniae*	Recurrent respiratory infections	Upper respiratory infection not requiring hospitalization
	Alopecia areata	Alopecia areata of the scalp, three patches, partial response to topical and systemic steroids	Alopecia areata	Smaller patches and fluctuating course with topical steroids
	Dysimmune hepatitis	Hepatitis with mild cholestasis and normal liver function. Anti-SM antibodies positivity and anti-LKM antibodies negativity. Histology: Rich portal and lobular lymphocytic infiltrate with occasional interface expression with CD3^+^ as predominant component.	Dysimmune hepatitis	Residual self-limiting sporadic increase in transaminase levels
	Splenomegaly	Spleen (US) 16 cm (bipolar diameter) with hypoechoic intraparenchymal areas with a maximum diameter of ∼19 mm	Splenomegaly	Spleen (US) 12 cm (bipolar diameter), heterogeneous echotexture due to the persistence of some hypoechoic areas with ill-defined margins, not clearly vascularized, and containing some hyperechoic components consistent with calcifications Spleen (MRI) 13 cm, with some vaguely hypointense nodular-like areas are observed on T2W
	Persistent lymphadenopathies	US (max dimensions) LTC lymph nodes L 21 × 9 mm, R 14 × 6 mm	Fluctuating lymphadenopathies	US (max dimensions) LTC lymph nodes R 15 × 6 mm; L 14 × 5 mm, reduced echogenicity and poor visualization of the hilum Submandibular lymph nodes R 20 × 6 mm; L 15 × 7 mm, regular echotexture Axillary lymph nodes R 15 × 3 mm; L 12 × 6 mm, regular echotexture Supra clavicular lymph nodes L 10 × 4 mm, regular echotexture MRI (max dimensions) LTC lymph nodes 2.3 cm Armpit lymph nodes 1.9 cm
	Enteritis	Alternating bowel habits with episodes of diarrhea EGDs: Macroscopically normal findings Colonoscopy: Findings normal except for aptha on the edge of the ileocecal valve Histology: Increased neutrophilic, eosinophilic, lympho-plasmacytic, and granulocytic inflammatory component partially aggressive with plasma cells. Numerous foamy histiocytes in the left colon. Marked increase in duodenal intraepithelial T cells. Complete intestinal metaplasia of the gastric antrum	Pulmonary interstitial disease	CT scan: multiple areas of air trapping with subsegmental distribution in the right lower lobe. Hypoventilation. Bilateral basal fibrotic hypoventilatory streaks Plethysmography: Air trapping with increased airway resistance. TLC: 2.72 L (78%), RV: 1.27 L (146%), RV/TLC: 44 (178%). FEV_1_/FVC 93% DLCO 4.77 (73%) for VA reduced (2.09, 58%). Kco slightly increased (2.28, 124%)
			Thymic hyperplasia	CT (diameter): 5 × 1.7 × 7 cm, cervical extension to the jugular veins, contrast enhancement
Hematological	Thrombocytopenia Persistent leukopenia Anemia	Worst value recorded PLT count, 124.000/mm^3^ WBC count, 2,020/mm^3^ Hb level, 9 g/dl	Thrombocytopenia Fluctuant leukopenia	Worst value recorded PLT count, 114.000/mm^3^ WBC count, 2,420/mm^3^
Immune evaluation	Major immunophenotype anomalies: B cells (CD19^+^) Reduced memory B Slightly expanded transitional B cell Slightly expanded CD21^low^ Senescent CD8	15% (124 cell/µl) 1% (39% non-switched) 10% 4.7% n.a.	Major immunophenotype anomalies: B cells (CD19^+^) Memory B cell Expanded transitional B cell Slightly expanded CD21^low^ Expanded senescent CD8	11% (120 cells/ul) 4% (89% non-switched) 20% 4.2% 13%
	IgG IgM IgA	934 mg/dl preinfusion in IRT 257 mg/dl 68 mg/dl	IgG IgM IgA	1,007 mg/dl 66 mg/dl 38 mg/dl
Medications	​	Dosage/frequency	​	Dosage/frequency
	SCIG replacement	15 *g*/mo	SCIG replacement	15 *g*/mo
	PEP mask	prescribed but not performed	PEP mask	prescribed but not performed
	TMP-SMX	160 mg/800 mg	TMP-SMX	160 mg/800 mg
	Folic acid	one tablet three times per wk	Folic acid	1 tablet three times per wk
	Phytomenadione	5 mg/day twice per wk	Phytomenadione	5 mg/day twice per wk
	Cholecalciferol 25,000 IU	One vial every 3 wk	Cholecalciferol 25,000 UI	1 vial every 3 wk
	​	​	Sirolimus	3.5 mg/day
	​	​	Testosterone	25 mg/mo intramuscular

Ab, antibody; DLCO, diffusing capacity of the lungs for carbon monoxide; EGDs, esophagogastroduodenoscopies; Hb, hemoglobin; FEV_1_, forced expiratory volume in 1 s; FVC, forced vital capacity; HHV6, human herpesvirus 6; IRT, immunoglobulin replacement therapy; Kco, carbon monoxide transfer coefficient; LKM, liver kidney microsome; L, left; LTC, laterocervical; PEP, positive expiratory pressure; PLT, platelet; R, right; RSV, respiratory syncytial virus; RV, residual volume; SCIG, subcutaneous immunoglobulin; SM, smooth muscle; TLC, total lung capacity; TMP-SMX, trimethoprim-sulfamethoxazole; UI, international units; US, ultrasound; VA, alveolar volume; WBC, white blood cell.

To address autoimmunity and lymphoproliferation, rapamycin was selected as a first-line treatment. Hepatic cytolysis and spleen size reduced, while cytopenias improved. Fluctuating lymphadenopathies, leukopenia, and spleen lesions persisted; thymic hyperplasia progressed. Despite the adequate rapamycin dose and plasma levels at the upper-end of therapeutic range, during rapamycin treatment, the patient developed severe, asymptomatic, infiltrative lung disease characterized by a restrictive pattern on lung function tests, reduced diffusing capacity, and evidence of interstitial involvement. Differential considerations, including *PRKCD*-related disease progression and potential hypersensitivity to rapamycin, precluded establishing the cause of lung involvement (24). Because the pulmonary involvement could not be clearly attributed to PRKCD-related progression versus drug toxicity, and because the patient’s sirolimus trough levels were already at the upper end of the therapeutic range, further dose escalation was not considered safe. Rapamycin treatment was therefore withdrawn due to lack of efficacy and/or potential causative role in lung disease.

Given ongoing multisystem disease activity and the need to prevent further organ damage, we considered several therapeutic alternatives to sirolimus. A steroid-sparing strategy was prioritized in a preadolescent patient experiencing significant growth restriction, delayed pubertal development, and immunodeficiency. B cell depletors were deemed unsuitable due to anecdotal association with disease relapse and lack of effectiveness in the patient’s brother. In addition, it is increasingly clear that prolonged B cell depletion is not without significant consequences beyond secondary hypogammaglobulinemia. This approach entails risks such as sustained B cell aplasia, which cannot be fully mitigated by Ig replacement, particularly in a preadolescent with PRKCD deficiency affecting both B and T cell compartments.

Mycophenolate mofetil and other broad immunosuppressants were considered suboptimal due to limited evidence of durable control in PRKCD deficiency and the risk of cumulative toxicity in a preadolescent with underlying immunodeficiency. HSCT was not pursued due to multiorgan involvement, the uncertain impact of the *PRKCD* variant on extra-hematopoietic tissues, and limited data on outcomes.

Compassionate use of leniolisib was based on a precision, pathway-guided rationale with multiple clinical, molecular, and laboratory indicators of PI3K/AKT/mTOR pathway hyperactivity (Fig. 1). The index patient’s clinical and immune phenotypes overlapped with APDS. His T cells exhibited ex vivo hyper-phosphorylation of S6—an established downstream surrogate marker of the PI3K/AKT/mTOR axis, analogous to pAKT and used here as a proxy for mTOR activation (25)—at levels comparable to those observed in APDS patients. These findings align with the partial clinical response observed during rapamycin treatment and highlighting the in vivo relevance of pathway hyperactivity. We observed ex vivo normalization of stimulated phospho-S6 (pS6) in patient CD4^+^ and CD8^+^ T cells after experimental addition of rapamycin or idelalisib (a selective PI3Kδ inhibitor). Research suggests that *PRKCD* variants impair the inhibitory function of PKCδ on the PI3K pathway, strengthening the use of PI3Kδ inhibition to target underlying immune dysregulation (5). We performed the same in vitro pS6 assay, with addition rapamycin and idelalisib, in the older sibling obtaining comparable results—again consistent with mTOR pathway hyperactivity and normalization of S6 phosphorylation to healthy-donor levels upon pharmacologic inhibition (Fig. 1 C). The sibling was not considered a suitable candidate for in vivo administration of leniolisib for the end-stage kidney disease, which may have unanticipated impact on pharmacokinetics and plasma concentrations.

**Figure 2.**
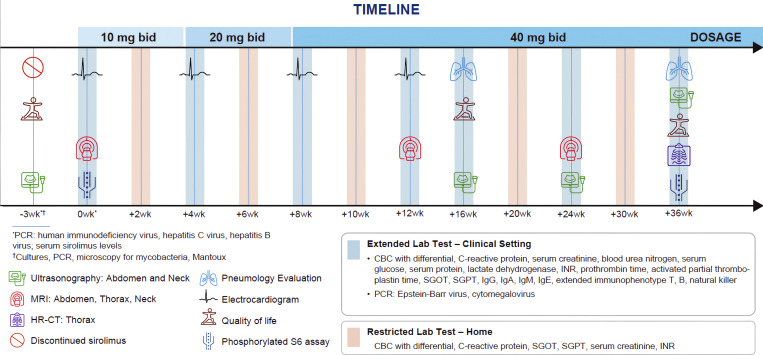
**Leniolisib treatment protocol.** Bid, twice daily; CBC complete blood cell count; INR, international normalized ratio; PCR, polymerase chain reaction; SGOT, serum glutamic-oxaloacetic transaminase; SGPT, serum glutamic-pyruvic transaminase.

Based on this rationale, leniolisib was administered according to a compassionate-use protocol in this 14-year-old patient (weight 34.8 kg) and titrated to a maintenance dose of 40 mg twice daily; details of dose escalation are provided in the supplementary material. At the time of writing, the patient has received leniolisib for a total of 10 mo. Fig. 2 depicts the timeline of leniolisib treatment. The patient did not experience serious adverse events. Nonserious adverse events commonly reported among patients with APDS receiving leniolisib such as infections, skin rashes, gastrointestinal symptoms, fatigue, neutropenia, and elevated liver enzymes were not observed.

Sustained disease control and multiparameter improvement were evident (Table 3). The patient engaged in academic, social, and athletic activities. Self-perceived quality of life improved. Neither infections requiring hospitalization nor mild recurrent infections were reported. One episode of self-limiting enteritis was reported after the observation period. Trimethoprim/sulfamethoxazole was discontinued 8 mo after leniolisib initiation. Alopecia areata resolved without the need for topical glucocorticoids. No new autoimmune manifestations were observed. Cervical and axillary lymphadenopathy resolved.

**Table 3.** Post-leniolisib treatment parameters

​	Details	T + 8 wk	T + 12 wk	T + 16 wk	T + 24 wk	T + 36 wk
Auxological parameters	Weight	35.5 kg	35.5 kg	36.2 kg	38 kg	38.2 kg
	Height	149.6 cm	n.a.	n.a.	154 cm	154 cm
QoL (SF-36), %	​	​	​	66%	​	82%
Hematological	Hb level	14.6 *g*/dl	14.4 *g*/dl	15.5 *g*/dl	15.8 *g*/dl	16.8 *g*/dl
	WBC count	5,240/mm^3^	4,970/mm^3^	4,120/mm^3^	4,600/mm^3^	5,060/mm^3^
	Lymphocyte count	1,938/mm^3^	2,160/mm^3^	1,830/mm^3^	2,120/mm^3^	2,300/mm^3^
	PLT count	158,000/mm^3^	169,000/mm^3^	165,000/mm^3^	150,000/mm^3^	192,000/mm^3^
Immunophenotype	CD3	1,656/mm^3^	1,866/mm^3^	1,541/mm^3^	1,594/mm^3^	1,435/mm^3^
	CD4	1,183/mm^3^	1,265/mm^3^	1,119/mm^3^	1,111/mm^3^	992/mm^3^
	CD8	429/mm^3^	551/mm^3^	401/mm^3^	466/mm^3^	424/mm^3^
	CD19	190/mm^3^	251/mm^3^	213/mm^3^	269/mm^3^	307/mm^3^
	CD16-56	122/mm^3^	193/mm^3^	145/mm^3^	180/mm^3^	204/mm^3^
	Naive B cell	180/mm^3^	241/mm^3^	200/mm^3^	261/mm^3^	298/mm^3^
	Memory B cell	6/mm^3^	10/mm^3^	10.7/mm^3^	13.3/mm^3^	12/mm^3^
	Transitional B cell	15%	15%	5.8%	4.7%	1.6%
	Naive CD4 cell	733/mm^3^	746/mm^3^	615/mm^3^	855/mm^3^	798/mm^3^
	Naive CD8 cell	197/mm^3^	28/mm^3^	180/mm^3^c	89/mm^3^	224/mm^3^
	IgG	682 mg/dl	686 mg/dl	769 mg/dl	-	979 mg/dl
	IgM	67 mg/dl	39 mg/dl	40 mg/dl	36 mg/dl	32 mg/dl
	IgA	31 mg/dl	26 mg/dl	25 mg/dl	24 mg/dl	30 mg/dl
Lung function	DLCO-Plethysmography	​	​	TLC: 2.88 L RV/TLC: 31% Reduction in air trapping, plethysmography improved	​	​
Radiological CT	Thymus	​	​	​	​	Volume reduction: 44.62%
	Lung	​	​	​	​	Resolution of consolidation bands and tree-in-bud appearance, less prominent “mosaic attenuation areas”
	Lymph nodes	​	​	​	​	The left axillary lymph node shows a reduction in size and postcontrast enhancement
Radiological MRI	LTC lymph nodes	​	Max 1.8 cm	​	Max 2.1 cm	​
	Axillary lymph nodes	​	n.a.	​	Max 1.3 cm	​
	Spleen	​	13 cm	​	12.5 cm (volume reduction of 52.37% vs. pre-leniolisib); the hypointense nodular area within the splenic parenchyma is no longer identifiable	​
Radiological US	LTC lymph nodes	​	<1 cm	​	<1 cm	​
	Submandibular lymph nodes	​	R 18 × 6 mm	​	R 12 × 4 mm L 13 × 5 mm	​
	Axillary lymph nodes	​	<1 cm	​	R 17 × 3 mm L 18 × 3 mm	​
	Subclavicular lymph nodes	​	<1 cm	​	<1 cm	​
	Spleen	​	12.3 cm	​	11 cm	​

DLCO, diffusing capacity of the lungs for carbon monoxide; Hb, hemoglobin; L, left; LTC, laterocervical; NA, not applicable; PLT, platelet; QoL, quality of life; R, right; RV, residual volume; SF-36, 36-Item Short Form Health Survey; T, time; TLC, total lung capacity; US, ultrasound; WBC, white blood cell.

**Figure 3.**
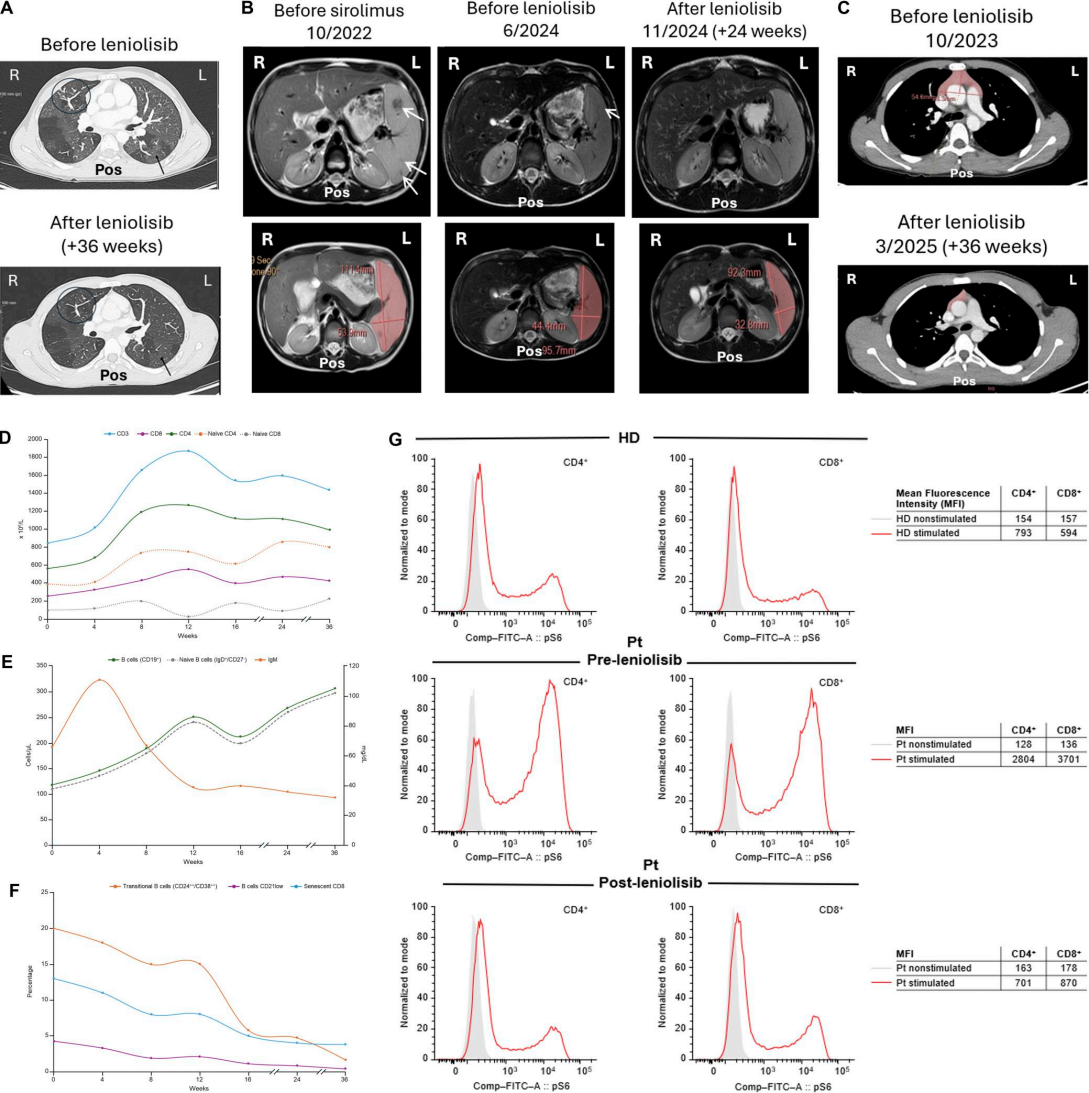
**Pulmonary, lymphoproliferation, immunophenotype, and pS6 changes. (A)** HRCT scans of the lungs before and after treatment with leniolisib. Treatment resolved consolidation streaks and the “tree-in-bud” pattern (circles). Mosaic attenuation areas persist but are less pronounced compared with pretreatment examination (arrows). **(B)** MRI scans of the spleen before sirolimus and leniolisib and after treatment with leniolisib. A nodular hypointense area in the splenic parenchyma visible in earlier scans (white arrows) was no longer detectable during treatment with leniolisib. **(C)** CT scans of the thymus before and after treatment with leniolisib. **(D–F)** Immunophenotyping during leniolisib treatment. **(D)** Absolute T cell subset counts. **(E)** Absolute counts of total and naive B cells and IgM levels. **(F)** Percentage of transitional B cells, CD21^low^ B cells, and senescent CD8^+^ T cells. **(G)** pS6 flow cytometry assay of stimulated and nonstimulated CD4^+^ and CD8^+^ T cells from HD (top), and patient pre- (middle), and posttreatment with leniolisib (bottom). Elevated pS6 decreased after treatment with leniolisib. Comp-FITC-A, compensation control fluorescein isothiocyanate; HD, healthy donor; L, left; MFI, mean fluorescence intensity; Pos, posterior; Pt, patient; R, right.

Lung volumes and alveolar diffusing capacity improved despite the patient never undergoing prescribed respiratory physiotherapy and current tobacco use. Computed tomography (CT) scans performed before and after treatment revealed favorable structural changes related to interstitial lung disease, reduction of bronchiectasis and air trapping, and resolution of the tree-in-bud pattern (Fig. 3 A). Magnetic resonance imaging (MRI) and ultrasonography showed a progressive reduction in the size of the spleen (52.4%; Fig. 3 B), lymph nodes, and thymus (44.6%; Fig. 3 C) (Table S1). Splenic lesions resolved, and structural homogeneity was maintained.

Cytopenias resolved, with hemoglobin and platelets levels and white blood cell counts remaining within reference ranges (Table 3).

At presentation, absolute circulating B cell counts were at the lower end of the age-adjusted range, in the context of global lymphopenia. Immunophenotyping showed expansion of transitional B cells, reduction of memory B cells, and expansion of CD21^low^ B cells, consistent with a block in B cell maturation. Serum IgM was elevated (hyper-IgM pattern). During treatment, T and B cell populations improved (Fig. 3, D–F and Table 3). CD3^+^, CD4^+^, and CD4^+^ naive T cells increased. The percentage and absolute counts of total and naive B cells increased. We observed an early decrease in transitional and CD21^low^ B cells together with a fall in serum IgM, suggesting a partial release of this maturational block along the B cell lineage. Memory B cells showed only minimal early increase, which is expected to require longer time to recover.

During treatment, pS6 levels in CD4^+^ and CD8^+^ T cells were comparable to healthy controls (Fig. 3 G). No differences in pS6 levels were detected in samples obtained 10 h (corresponding to half-life of leniolisib) or 1 h (corresponding to maximum plasma concentration) after leniolisib intake, suggesting stable normalization of mTOR activity between doses.

This was the first compassionate use of leniolisib for the treatment of an IEI other than APDS (26). Leniolisib was well tolerated and improved clinical and laboratory parameters that were only partially controlled with rapamycin. Quality of life also improved: fatigue, infections, lymphoproliferation, or cytopenias were not reported after initiating leniolisib. The patient did not require corticosteroids, B cell depletors, or additional antibiotic treatment while receiving leniolisib.

Lung performance improved, with enhancements in lung volumes and alveolar diffusing capacity. The absence of complete disease reversal after rapamycin withdrawal suggests that pulmonary involvement was due to underlying disease.

Spleen size reduction and improvement in splenic structural changes were observed; the latter was present during rapamycin therapy. Episodic, persistent lymph node enlargements, which continued with rapamycin treatment, resolved during leniolisib treatment. Blood cell counts, which previously fluctuated during rapamycin therapy, normalized. Leniolisib treatment improved immunophenotype abnormalities and was consistent with outcomes reported among patients with APDS (21, 22, 23). mTOR hyperactivity (elevated pS6) in CD4^+^ and CD8^+^ T cells reduced to healthy control levels following treatment.

## Discussion

The molecular mechanisms underlying the impact of leniolisib in *PRKCD* deficiency and the precise interaction between PI3K and PKCδ require further elucidation. While findings from a single patient cannot be generalized, this experience supports the use of PI3Kδ inhibitors for the treatment of relevant IEIs besides APDS.

Clinical trials in APDS utilize a prescribed weight-based dosing strategy, but it is unknown if this dosing strategy is optimal in disorders such as *PRKCD* deficiency. Thus far, our patient has maintained a good response to 40 mg twice daily, but adjustments over time to account for growth and observed degree of clinical response will likely be needed.

One limitation of working with ultra-rare diseases is challenges compiling large cohorts of patients. International collaboration to identify cases is warranted to determine efficacious therapeutic strategies. Comprehensive selection of patients based on clinical, genetic, immunophenotypic, and molecular features—particularly those associated with AKT/mTOR pathway hyperactivity—will optimize efficacy and safety. Trials using selected patient populations may help establish leniolisib as an alternative to mTOR inhibitors, particularly in cases of inadequate disease control or adverse effects. Long-term monitoring will be essential to evaluate the overall safety and efficacy of leniolisib.

This case highlights the capability of leniolisib to address immunodeficiency and lymphoproliferation associated with *PRKCD* deficiency, paving the way for broader clinical applications in other IEIs and multifactorial immune-mediate diseases. In the era of computational drug repurposing, exploring the application of existing drugs for rare diseases is essential. This case supports the notion that healthcare professionals should rationally explore the application of exiting molecules for diseases where treatments are lacking or nonexistent.

## Materials and methods

### Treatment protocol and outcomes

Treatment protocol details are presented in Table S2, including dose-escalation strategy; clinical, laboratory, and imaging evaluations related to safety monitoring; and outcome achievements. Compassionate use of leniolisib was approved on April 26, 2024, by the Regional Pediatric Local Ethics Committee at Meyer Children’s Hospital, Florence. Both the legal representative and patient provided written informed consent before treatment initiation. Pharming Group N.V. provided free access to leniolisib for compassionate use.

### Safety

Safety was assessed throughout the intervention period to monitor adverse events and ensure timely management. Adverse events were classified as serious, of special interest, or nonserious, with predefined thresholds for reporting and intervention. The “expected” adverse events (consistent with safety profile listed in prescribing information of leniolisib) included infections, gastrointestinal symptoms, skin manifestations, cytopenia, and elevation of liver enzymes. If safety thresholds were exceeded, adjustments or discontinuation of treatment were planned.

### Treatment outcomes

Primary outcomes were selected to assess therapeutic impact on disease and patient health, based on clinical, imaging, and laboratory evaluations.

Key metrics:

#### Clinical parameters

Assessment of overall health status and health-related quality of life (36-Item Short Form Health Survey) (27), incidence of infections, absence of progression in preexisting autoimmune-associated manifestations (e.g., no increase in hepatic cytolysis markers, no extension of alopecia lesions, and no exacerbation of gastrointestinal involvement), assessment of newly emerging autoimmune phenomena, incidence of noninfectious lymphadenopathy episodes, and lung function assessment, including lung volumes and alveolar diffusing capacity.

#### Imaging parameters

Longitudinal assessment of lung, spleen, lymph nodes, and thymus size, along with characterization of structural abnormalities, evaluated through ultrasound, MRI (Achieva 3 Tesla, Philips) or high-resolution CT (HR-CT; TC Revolution, GE Medical Systems). Lung HR-CT was acquired using a lung parenchyma window with consistent acquisition and reconstruction parameters. Spleen MRI was acquired via T2-weighted Turbo Spin Echo sequence in the axial plane. Spleen volumetry was calculated using the IntelliSpace Portal (Philips). CT of the thymus was acquired using a mediastinal window inspiration. Thymus volumetry was calculated using the IntelliSpace Portal (Philips). CT of lymph nodes was acquired using a mediastinal window.

#### Hematologic parameters

Changes in hemoglobin concentration (g/dl), platelet count (×10^3^/μl), absolute white blood cell count (×10^3^/μl), and lymphocyte count (cells/μl).

#### Immunological parameters

Variations in the percentage and absolute number of naive B and T cells; transitional B cells and senescent T cells (percentage; cells/μl); plasma concentrations of IgM and IgA with IgG replacement therapy (mg/dl); variations in stimulated pS6 in CD4^+^ and CD8^+^ T cells (geometric mean of mean fluorescence intensity). Reference ranges for immune subsets and IgM were age-matched and obtained from literature (28, 29, 30, 31). Ranges were as follows: B cells (CD19^+^), 226–370 cells/μl; naive B cells (CD27^−^IgD^+^), 171–293 cells/μl; transitional B cells (CD24^++^CD38^++^), 10–30 cells/μl −3.9 to 7.8%; CD21^low^ B cells (CD21^low^CD38^low^), 2–10 cells/μl −0.9 to 3.3%; CD3^+^ T cells, 954–2,332 × 10^6^/L; CD4^+^ T cells, 610–1,446 × 10^6^/L; CD8^+^ T cells, 282–749 × 10^6^/L; naive CD4^+^ T cells (CD45RA^+^CCR7^+^CD4^+^), 230–770 × 10^6^/L; naive CD8^+^ T cells (CD45RA^+^CCR7^+^CD8^+^), 240–710 × 10^6^/L; senescent CD8^+^ T cells (CD57^+^CD8^+^), 10–40 cells/μl −1.83 to 25%; and IgM, 42.4–197 mg/dl.

### pS6 assay

Peripheral blood mononuclear cells (PBMCs, 2 × 10^6^) were plated in 96-well plates at the concentration of 2 × 10^5^ cells per well in 200 μl of RPMI overnight at 37°C. For the stimulation, the plate was precoated with Mouse Anti-CD3 human (10 µg/μl) (86022706; Sigma-Aldrich) in 100 μl phosphate-buffered saline for 2 h. After dispensation of cells, 1 μl of Mouse Anti-Human CD28 (Purified NA/LE Mouse Clone CD28.2 [RUO], 555725, BD Biosciences) was added to each well.

After 24 h, PBMCs from the patient and a healthy control were stained for Mouse Anti-Human CD4 APC (555349; BD Pharmingen), Mouse Anti-Human CD8 PerCP-Cy5.5 (Clone SK1, 341050; BD Biosciences), and Rabbit Anti-Human Phospho-S6 Ribosomal Protein Antibody (Ser235/236) (BK2211LCST, Cell Signaling Technology). For secondary staining, Goat anti-Rabbit Ig Human ads-FITC antibody (Cat. No: 4010-02; Southern Biotech) was utilized. Cal101 and rapamycin were added before stimulation and used as inhibitors for PI3Kδ and mTOR, respectively, as previously described (32). All samples were collected with a FACSCanto flow cytometer and analyzed with FlowJo software. Data were analyzed using a two-sided independent samples Kruskal–Wallis test with Bonferroni correction.

### Online supplemental material

Table S1 presents longitudinal spleen and thymus volume measurements over time, while Table S2 details the treatment regimen and the associated monitoring and follow-up plan.

## Data availability

The data presented in this article are not readily available because of ethical and privacy restrictions. Requests to access the dataset should be directed to the corresponding author. Fig. 1 A was modified using https://BioRender.com.

## Supplementary Material

Table S1 shows the organ volume over time.

Table S2 shows the description of treatment and monitoring plan.
