# Supplementary material for: Expanding the scope of PI3K-δ inhibition: Leniolisib treatment in PRKCD deficiency
Source: J Hum Immun. 2026 Apr 20;2(3):e20250203. doi: 10.70962/jhi.20250203 (PMC13177475; doi:10.70962/jhi.20250203)
Supplement: Table S1 — shows the organ volume over time. [file jhi_20250203_tables1.docx]

**Table S1: Organ volume over time.**

| **Time point** | **Before Sirolimus** | **Before Leniolisib** | **24-36 Weeks After Leniolisib** |
| --- | --- | --- | --- |
| Spleen 3D volume, cm^3^ | 610.868 | 342.973 | 290.952 |
| Thymus 3D volume, cm^3^ | _ | 39.541 | 21.896 |
