# Supplementary material for: Expanding the scope of PI3K-δ inhibition: Leniolisib treatment in PRKCD deficiency
Source: J Hum Immun. 2026 Apr 20;2(3):e20250203. doi: 10.70962/jhi.20250203 (PMC13177475; doi:10.70962/jhi.20250203)
Supplement: Table S2 — shows the description of treatment and monitoring plan. [file jhi_20250203_tables2.docx]

**Table S2: Description of treatment and monitoring plan.**

| **Timepoint** | **Assessments** |
| --- | --- |
| **T -3 w** | Discontinue sirolimus   - Mycobacterial culture, PCR, and smear from sputum - Mantoux test - Abdomen and neck US - Informed consent - QoL questionnaires |
| **T 0 w** | Initiate leniolisib 10 mg twice daily   - CBC w/ differential, CRP, sCr BUN, sGlucose, IgG, IgA, IgM, IgE, serum protein, LDH, INR, PT, aPTT, SGOT, SGPT - Extended immunophenotyping - PCR for EBV, CMV, HIV, HCV, HBV, serum sirolimus levels - pS6 (referral lab) - Abdomen, thorax, neck MRI - Electrocardiogram (ECG) |
| **T +2 w** | - CBC w/differential, CRP, SGOT, SGPT, sCrea, INR |
| **T +4 w** | Increase leniolisib to 20 mg twice daily   - CBC w/ differential, CRP, sCr, BUN, sGlucose, IgG, IgA, IgM, IgE, serum protein, LDH, INR, PT, aPTT, SGOT, SGPT - Extended immunophenotyping - PCR for EBV, CMV - ECG |
| **T +6 w** | - CBC w/differential, CRP, SGOT, SGPT, sCrea, INR |
| **T +8 w** | Increase Leniolisib to 40 mg twice daily   - CBC w/ differential, CRP, sCr, BUN, sGlucose, IgG, IgA, IgM, IgE, serum protein, LDH, INR, PT, aPTT, SGOT, SGPT - Extended immunophenotyping - PCR for EBV, CMV - ECG |
| **T +10 w** | - CBC w/differential, CRP, SGOT, SGPT, sCrea, INR |
| **T +12 w** | - CBC w/differential, CRP, sCr, BUN, sGlucose, IgG, IgA, IgM, IgE, serum protein, LDH, INR, PT, aPTT, SGOT, SGPT - Extended immunophenotyping - PCR for EBV, CMV - Abdomen, thorax, neck MRI - ECG |
| **T +16 weeks** | - CBC w/differential, CRP, sCr, BUN, sGlucose, IgG, IgA, IgM, IgE, serum protein, LDH, INR, PT, aPTT, SGOT, SGPT, - Extended immunophenotyping - PCR for EBV, CMV - Abdomen and neck US - Pulmonary evaluation (with lung volumes) - QoL questionnaires |
| **T +20 weeks** | - CBC w/differential, CRP, SGOT, SGPT, sCr, INR |
| **T +24 weeks** | - CBC w/differential, CRP, sCr, BUN, sGlucose, IgG, IgA, IgM, IgE, serum protein, LDH, INR, PT, aPTT, SGOT, SGPT - Extended immunophenotyping - PCR for EBV, CMV - US abdomen and neck - Abdomen, thorax, neck MRI |
| **T +30 weeks** | - CBC w/differential, CRP, SGOT, SGPT, sCrea, INR |
| **T +36 weeks** | - CBC w/differential, CRP, sCr, BUN, sGlucose, IgG, IgA, IgM, IgE, serum protein, LDH, INR, PT, aPTT, SGOT, SGPT - Extended immunophenotyping - PCR for EBV, CMV - pS6 (referral lab) - HRCT thorax - US abdomen and neck - DLCO and plethysmography - QoL questionnaires. |

aPTT, activated partial thromboplastin time; BUN, blood urea nitrogen; CBC, complete blood count; CMV, cytomegalovirus; CRP, C-reactive protein; DLCO, diffusing capacity of the lungs for carbon monoxide; EBV, Epstein-Barr virus; ECG, electrocardiogram; SGOT, serum glutamic-oxaloacetic transaminase; SGPT, serum glutamic-pyruvic transaminase; HBV, hepatitis B virus; HCV, hepatitis C virus; HIV, human immunodeficiency virus; HRCT, high-resolution computed tomography; Ig, immunoglobulin; INR, international normalized ratio; LDH, lactate dehydrogenase; MRI, magnetic resonance imaging; PCR, polymerase chain reaction; pS6, phosphorylated S6; PT, prothrombin time; QoL, quality of life; sCr, serum creatinine; sGlucose, serum glucose; T, time; US, ultrasound.
